# CICERO: a versatile method for detecting complex and diverse driver fusions using cancer RNA sequencing data

**DOI:** 10.1186/s13059-020-02043-x

**Published:** 2020-05-28

**Authors:** Liqing Tian, Yongjin Li, Michael N. Edmonson, Xin Zhou, Scott Newman, Clay McLeod, Andrew Thrasher, Yu Liu, Bo Tang, Michael C. Rusch, John Easton, Jing Ma, Eric Davis, Austyn Trull, J. Robert Michael, Karol Szlachta, Charles Mullighan, Suzanne J. Baker, James R. Downing, David W. Ellison, Jinghui Zhang

**Affiliations:** 1 Department of Computational Biology, St. Jude Children’s Research Hospital, 262 Danny Thomas Place, Memphis, TN 38105 USA; 2 Pediatric Translational Medicine Institute, Shanghai Children’s Medical Center, Shanghai Jiao Tong University School of Medicine, Shanghai, China; 3 Department of Pathology, St. Jude Children’s Research Hospital, 262 Danny Thomas Place, Memphis, TN 38105 USA; 4 Department of Developmental Neurobiology, St. Jude Children’s Research Hospital, 262 Danny Thomas Place, Memphis, TN 38105 USA

**Keywords:** Gene fusion, Precision oncology, Fusion visualization, RNA-seq, Cloud computing

## Abstract

To discover driver fusions beyond canonical exon-to-exon chimeric transcripts, we develop CICERO, a local assembly-based algorithm that integrates RNA-seq read support with extensive annotation for candidate ranking. CICERO outperforms commonly used methods, achieving a 95% detection rate for 184 independently validated driver fusions including internal tandem duplications and other non-canonical events in 170 pediatric cancer transcriptomes. Re-analysis of TCGA glioblastoma RNA-seq unveils previously unreported kinase fusions (KLHL7-BRAF) and a 13% prevalence of EGFR C-terminal truncation. Accessible via standard or cloud-based implementation, CICERO enhances driver fusion detection for research and precision oncology. The CICERO source code is available at https://github.com/stjude/Cicero.

## Background

Gene fusions resulting from genomic structural variations (SVs), such as translocations, deletions, tandem duplications, and inversions in coding or regulatory regions, can be cancer-initiating events. Diverse types of gene fusions can lead to abnormal function or aberrant transcription of cancer driver genes. For example, activation of kinase and cytokine receptor signaling can be achieved by formation of chimeric transcripts merging exons of two partner genes (e.g., BCR-ABL in leukemia [1]), internal tandem duplication (ITD) in the juxtamembrane domain or kinase domain (e.g., FLT3 ITD in leukemia [2], FGFR1 ITD in brain tumors [3]), C-terminal truncation (e.g., EGFR in brain tumors [4] and MAP3K8 in melanoma [5]), promoter swapping (e.g., P2RY8-CRLF2 in leukemia [6]), or enhancer hijacking (e.g., IGH-EPOR in leukemia [7]). Gene fusions can define cancer subtypes and form an important class of therapeutic targets [8–10].

**Fig. 1:**
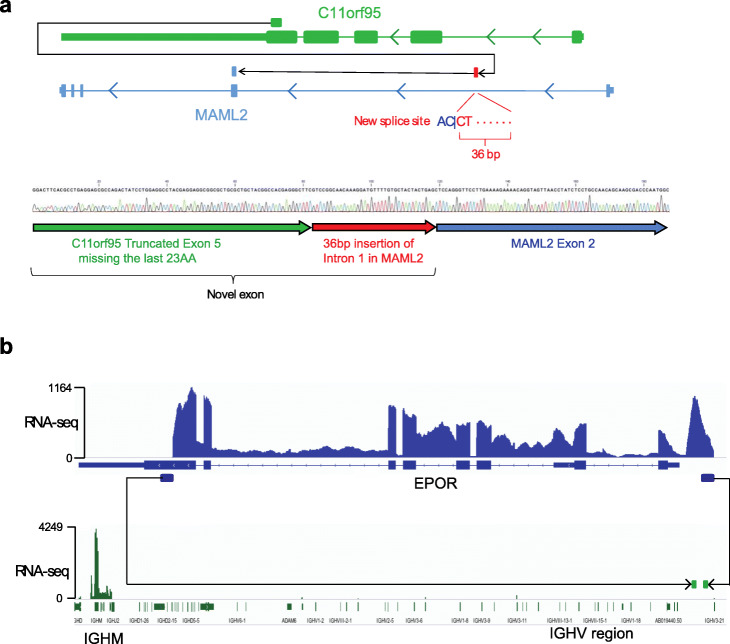
Examples of complex fusion cases missed by commonly used fusion detection tools. **a** A 3-segment C11orf95-MAML2 fusion in an ependymoma (SJEPD001509_D). The fusion breakpoints are shown at the top, which introduces a new splice site (reverse complement sequence AG|GT) within intron 1 of MAML2 (red arrow at the top). This resulted in replacing the last 23AA of C11orf95 with a 36 bp in-frame insertion, which was confirmed by Sanger sequencing shown at the bottom. This fusion can be detected by FusionCatcher but not the other three public methods. **b** IGH-EPOR fusion in a B-ALL (SJBALL020824_D1) which caused the insertion of EPOR gene into the highly repetitive IGH locus. *Y*-axis shows the coverage of RNA-seq at the two loci with arrows denoting the fusion breakpoints. None of the four public methods can detect this fusion

Paired-end short-read sequencing of transcriptomes (termed RNA-seq in the present study) has become a popular approach for fusion detection [11–13]. Various computational methods have been developed, leading to the discovery of many novel gene fusions in recent years [14]. However, when comparing gene fusions detected by RNA-seq with structural variations discovered by whole-genome sequencing [15], we recognized several limitations of existing RNA-seq analysis methods. Specific problems leading to false negatives were insertion of non-template sequence at fusion junctions [7]; use of cryptic/non-canonical exons; rearrangements within repetitive regions such as the immunoglobulin loci, where an enhancer can be juxtaposed to an oncogene; rearrangements internal to a single gene; and gene fusions with low transcription levels and generally high false positive prediction rates. Two examples of complex driver fusions missed by popular algorithms such as defuse [16], ChimeraScan [17], FusionCatcher [18], and STAR-Fusion [19] are shown in Fig. 1. The first is C11orf95-MAML2, a driver fusion formed by a novel exon which joins a truncated exon 5 of C11orf95 with 36 bp of intron 1 of MAML2 in a supratentorial ependymoma [20]. The second is an IGH-EPOR fusion, targetable by the JAK inhibitor ruxolitinib, with the fusion breakpoint occurring in a highly repetitive IGH locus in a B cell acute lymphoblastic leukemia (B-ALL) [21, 22].

To overcome these limitations, we developed CICERO (*C*ICERO *I*s *C*lipping *E*xtended for *R*NA *O*ptimization), a fusion gene detection algorithm which takes advantage of the increased next-generation sequencing (NGS) read length of current platforms to assemble RNA-seq reads bearing aberrant mapping signatures. The use of local assembly coupled with additional heuristics implemented to remove transcriptional artifacts enables the detection of diverse types of gene fusions at high sensitivity and accuracy. We show that CICERO is able to achieve high accuracy in analyzing a benchmark data set of 170 pediatric leukemia, solid tumor, and brain tumor transcriptomes and can enhance our ability to detect different types of driver gene fusions beyond the canonical chimeric exon-to-exon fusion transcripts in both pediatric and adult cancers. To further improve accuracy, gene fusions predicted from a single or a cohort of cancer transcriptomes can be curated in FusionEditor, an interactive viewer allowing inspection of protein domains involved in the fusion and evaluation of gene expression status in a fusion-positive sample. The source code is available from https://github.com/stjude/Cicero and a cloud-based implementation enables users to perform rapid RNA-seq fusion analysis via either point-and-click or command-line interfaces at https://platform.stjude.cloud/tools/rapid_rna-seq.

## Results

### Design of CICERO

**Fig. 2:**
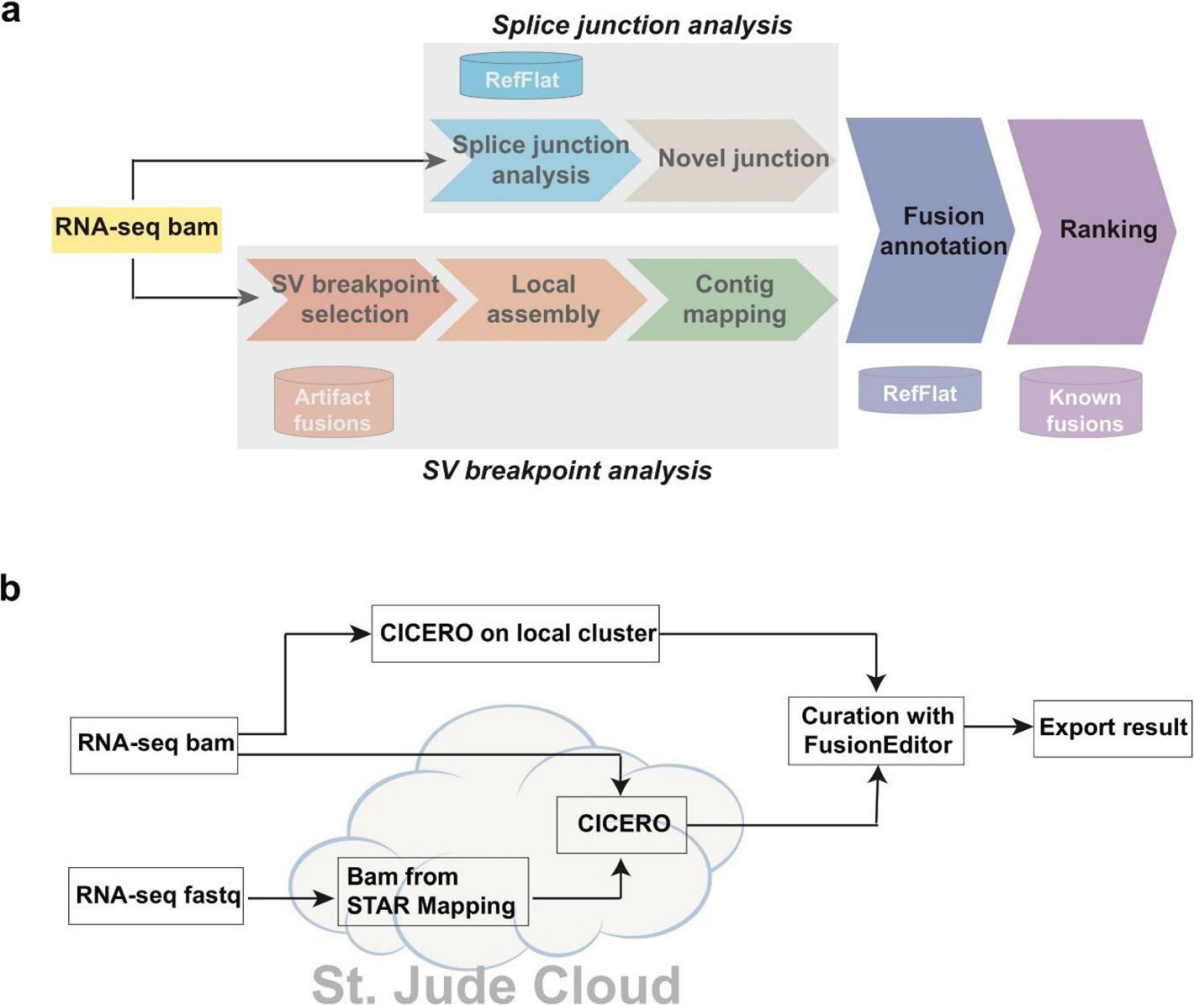
Fusion detection using CICERO. **a** Overview of CICERO algorithm which consists of fusion detection through analysis of candidate SV breakpoints and splice junction, fusion annotation, and ranking; key data sets used in each step are labeled. **b** Workflow of fusion detection. A user can submit an aligned BAM file or a raw fastq file as the input on a local computer cluster or on St. Jude Cloud. The raw output can be curated using FusionEditor and final results can be exported as a text file

To discover the diverse types of driver gene fusions in cancer, the overall design of CICERO is to integrate RNA-seq mapping with genomic features. This was implemented through the three key steps outlined in Fig. 2a: (1) fusion detection by de novo local assembly at candidate breakpoints and analysis of splice junction reads, (2) fusion annotation including a reading frame check for the fusion partner genes, and (3) ranking of candidate fusions based on the supporting evidence in RNA-seq and matches to known fusions. CICERO can be run from a local cluster or on St. Jude Cloud (https://platform.stjude.cloud/tools/rapid_rna-seq) which provides easy access via either an interactive point-and-click interface or the command-line for submitting batch jobs. More importantly, the Cloud pipeline effectively manages the burst of computing required for genome-wide mapping to assess uniqueness of each candidate fusion. This enables completion of the entire workflow on the Cloud, from RNA-seq mapping to fusion detection within hours, even for cases with massive numbers of rearrangements. Predicted gene fusions can then be imported to FusionEditor for manual curation and the curated file can be exported as the final results (Fig. 2b).

### Manual curation with FusionEditor

**Fig. 3:**
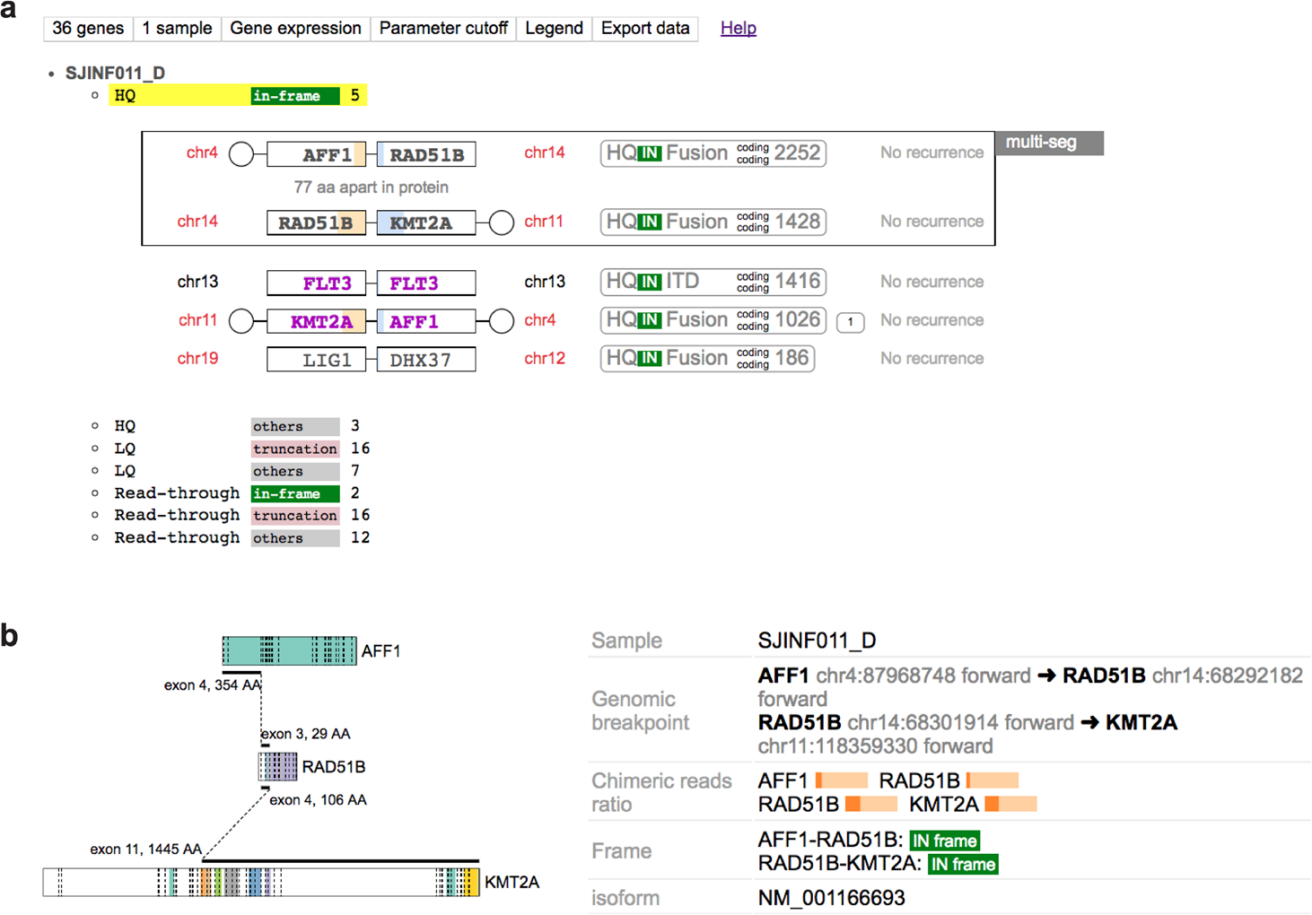
Visualization interface of FusionEditor for curating fusions predicted from one sample. **a** Table view which shows the five “HQ” (high quality) in-frame fusions predicted in an infant ALL (SJINF011_D). A 3-gene fusion involving AFF1-RAD51B-KMT2A is recognized automatically and marked by a box labeled as “multi-seg.” The reciprocal KMT2A-AFF1 fusion was also identified as a HQ in-frame fusion. Inter-and intra-chromosomal fusions are labeled with red and black text, respectively. Known fusions are labeled with purple text (e.g., KMT2A-AFF1 and FLT3 ITD in this case). **b** Graphical view depicting the breakpoints on the protein domains of the three partner genes. Additional information such as the chimeric reads ratio for each fusion breakpoint is shown to support assessing the validity of each predicted fusion

FusionEditor, an extension of our visualization tool ProteinPaint [23], imports CICERO output generated from one or multiple samples into an interactive browser (https://proteinpaint.stjude.org/FusionEditor/) to support manual curation. Within each sample, the predicted fusions are listed by quality grade—including high-quality (HQ), low-quality (LQ), or read-through—and annotated with in-frame/truncation status so that a user can prioritize curation of high-confidence calls while retaining the ability to review all predicted fusions (Fig. 3).

Each fusion can be viewed graphically (Fig. 3, Additional file 1: Figure S1) which shows the exon and amino acid position of the breakpoint at each partner gene or locus with respect to the reference gene model. This allows for manual appraisal of protein domains retained within the fusion protein. FusionEditor can also render breakpoints within UTR regions (Additional file 1: Figure S2), as well as promoter or intergenic fusions for review of enhancer hijacking events (Additional file 1: Figure S3). Complex fusions involving ≥ 3 partners can also be identified and visualized (e.g., AFF1-RAD51B-KMT2A fusion in Fig. 3). A user can edit fusion attributes by changing quality grade and fusion type, and by joining multiple breakpoints into a multi-segment fusion, or vice versa. The final curated results can be exported as a flat file for downstream analysis.

The interface for examining CICERO outputs from multiple samples enables quick identification of recurrent gene fusions in a cancer cohort (Additional file 1: Figure S1). Specifically, the recurrence of each gene fusion is summarized in a table (Additional file 1: Figure S1b) along with the assigned quality grade. A user can also search for fusions involving a specific gene, e.g., all fusions involving *TERT* (as shown in Additional file 1: Figure S1c). To support further evaluation of enhancer hijacking events, users may upload gene expression values (e.g., FPKM) from a cohort for inspection of aberrantly high expression in the selected fusion-positive sample (Additional file 1: Figure S1d). Via a point-and-click interface, the user can access additional details such as breakpoint position, domain information, soft-clipped read count, and gene expression level in the cohort (Additional file 1: Figure S1d and e).

### Comparison of CICERO with other methods of detecting somatic gene fusions

**Fig. 4:**
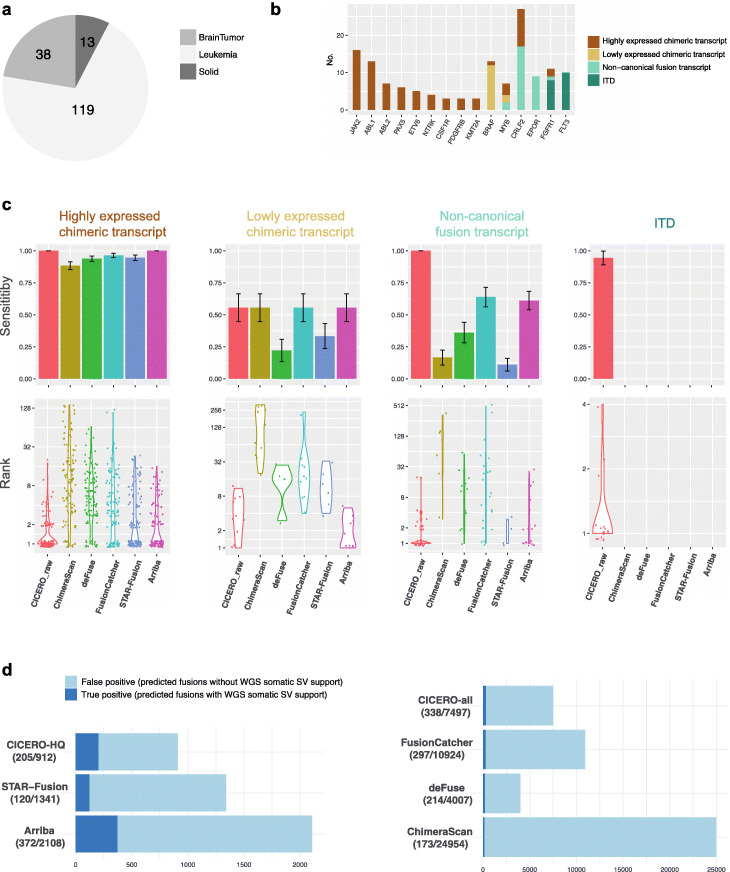
Comparison of CICERO with other methods on driver fusion detection. **a** Distribution of leukemia, solid tumor, and brain tumor in the 170 RNA-seq used for benchmark test. **b** Prevalence of recurrent (≥ 3) gene fusions in the benchmark data sets stratified by the following four classes: chimeric transcript caused by exon-to-exon fusion expressed at high (> 5 FPKM) or low level, internal tandem duplication (ITD), and other non-canonical fusions involving intronic or intergenic regions. **c** Comparison of the sensitivity (top panel) and ranking of the driver fusions among all predicted fusions (bottom panel) by CICERO and five other methods (ChimeraScan, deFuse, FusionCatcher, STAR-Fusion, and Arriba) in the four categories of driver fusion. The ranking by CICERO, labeled CICERO_raw, is based on fusion score alone without incorporating matches to known fusion status. Error bars representing standard deviation of detection sensitivity at the top panel were calculated by bootstrapping of samples with 100 iterations. **d** True positives (dark blue) and false positives (light blue) of predicted somatic fusions identified by CICERO and other fusion detection programs. The exact number of events is marked as (true positive/total prediction) under the name of each method. CICERO’s high-quality predictions are compared to those of STAR-fusion and Arriba (left panel) while all CICERO predictions are compared to those of FusionCatcher, deFuse, and ChimeraScan (right panel)

The benchmark data set consists of 184 driver gene fusions discovered in 170 samples of leukemia (*n* = 119), solid tumor (*n* = 13), and brain tumor (*n* = 38) (Fig. 4a, Additional file 2: Table S1 and S2) [3, 15, 21]. These 184 gene fusions, affecting well-characterized oncogenes in pediatric cancer (Fig. 4b), were orthogonally validated by paired tumor-normal WGS, capture sequencing, RT-PCR, and/or FISH. They therefore serve as a good benchmark standard for driver fusion detection, the most common use case for fusion detection using RNA-seq. The driver fusions can be classified into 4 categories based on genomic features and expression status: (1) highly expressed chimeric exon-to-exon fusions with FPKM > 5 for the N terminus partner gene (*n* = 112); (2) lowly expressed chimeric fusions (*n* = 18); (3) non-canonical fusions (*n* = 36), defined by one of the fusion breakpoints being in a non-coding region and representing mostly enhancer hijacking events; and (4) ITDs (*n* = 18).

We compared CICERO’s performance with five popular fusion detection methods: ChimeraScan [17], deFuse [16], FusionCatcher [18], STAR-Fusion [19], and Arriba [24]. All these methods produce large numbers of predictions that include true gene fusions as well as false positives caused by mapping ambiguity in repetitive regions, transcriptional read-through [25, 26], and other artifacts. We therefore evaluated performance based on sensitivity of detection, the ranking of driver fusions among all predictions per algorithm (Additional file 2: Table S2), and the false positive rate of all predicted fusions. To ensure a fair comparison, we used the CICERO ranking based on the fusion score alone (denoted CICERO_raw) which does not incorporate the knowledge-based quality grade. Events tagged as read-through are considered artifacts and thus excluded in all methods except for Arriba, as read-through events tagged by Arriba contain highly expressed oncogenic fusions (e.g., PR2Y8-CRLF2).

CICERO detected 95% of the driver fusions with an average ranking of 1.9, whereas ChimeraScan, deFuse, FusionCatcher, STAR-Fusion, and Arriba detected only 63%, 66%, 77%, 63%, and 78% with an average ranking of 37.0, 9.0, 18.1, 4.4, and 2.9, respectively (Additional file 2: Table S2). In the category of canonical exon-to-exon chimeric fusion, the detection rate is generally high across all methods for highly expressed fusions (ranging 88–100%, Fig. 4c) but low for the lowly expressed chimeric fusions (ranging 22–56%, Fig. 4c). In the category of non-canonical fusions, CICERO detected all 36 events, while the other methods (ChimeraScan, deFuse, FusionCatcher, STAR-Fusion, Arriba) detected 6, 13, 23, 4, and 22, respectively (Fig. 4c). In three cases, driver fusions such as IGH-EPOR and IGH-CRLF2 were detected exclusively by CICERO (Additional file 2: Table S2). Of the 18 ITD events in FLT3 or FGFR1, CICERO was able to detect 17 (Fig. 4c). None of the other five methods support ITD detection.

To evaluate the false positive rate of all predicted fusions, we considered RNA fusions that match somatic structural variations derived from paired tumor-normal DNA WGS data, available for 80 samples, as true positives (Methods). The detected driver fusions are all classified as high-quality by CICERO; therefore, we compared high-quality predictions by CICERO with calls from STAR-Fusion and Arriba, as these methods predicted relatively few gene fusions. Fusion predictions from ChimeraScan, deFuse, and FusionCatcher were compared with all CICERO calls. For high-quality predictions, the false positive rate of CICERO, STAR-fusion, and Arriba is 78%, 91%, and 82%, respectively (Fig. 4d, left panel; Additional file 3). When considering both high- and low-quality predictions, the false positive rate of CICERO, FusionCatcher, deFuse, and ChimeraScan is 95%, 97%, 95%, 99%, respectively (Fig. 4d, right panel; Additional file 3).

### CICERO analysis of adult cancer RNA-seq

We ran CICERO followed by manual curation using FusionEditor on RNA-seq data from The Cancer Genome Atlas Glioblastoma Multiforme (TCGA-GBM) which included 167 adult GBM patient samples (Additional file 2: Table S3) and compared the results to the gene fusions reported by the TCGA Research Network [27]. We focused on the gene fusions that had at least one partner gene included in COSMIC’s cancer gene census [28]. Of the 40 cancer gene-related fusions reported by TCGA, CICERO detected 33, including EGFR-SEPT14, FGFR3-TACC3, and NAA30-TERT (Additional file 2: Table S4).

**Fig. 5:**
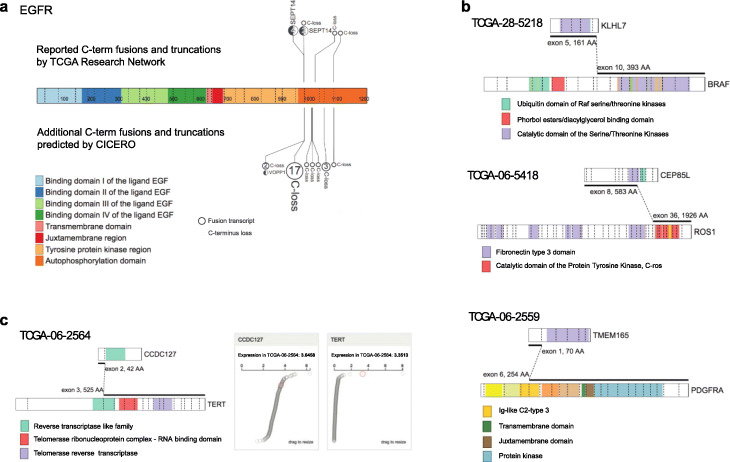
Examples of additional fusions identified by CICERO from TCGA-GBM cohort. The protein domain of the cancer gene involved in a fusion is labeled by colored legend. **a** Comparison of gene fusions leading to truncation of EGFR C terminal autophosphorylation domain discovered only by CICERO with those reported by both CICERO and the TCGA Research Network. Sites marked as “Closs” refer to out-of-frame C-terminal truncation fusions while those marked with a gene symbol refer to in-frame fusions. **b** Gene fusions that are likely to cause kinase activation. For the KLHL7-BRAF fusion, we selected the protein encoded by the KLHL7 short isoform NM_001172428 because the fusion breakpoint occurred at the last exon unique to this transcript. **c** CCDC127-TERT fusion in TCGA-06-2564 leading to over-expression of TERT. Right panel shows the FPKM value of CCDC127 and TERT of the entire GBM cohort with the red dot marking the fusion sample TCGA-06-2564

An additional 141 cancer gene-related fusions detected by CICERO were not reported by the TCGA Research Network, 60 of which involved *EGFR*, one of the most frequently mutated genes in GBM [27]. The additional EGFR fusions included one ITD (TCGA-27-2523) duplicating the tyrosine kinase domain (TKD) encoded by exons 18–25, matching a previously reported TKD duplication in two glioma cell lines [29]. The remaining EGFR fusions arise from intra-chromosomal rearrangements in regions 70Kb to 30 Mb away from EGFR or inter-chromosomal translocations (Additional file 2: Table S5). The most prevalent event, totaling 39 fusions in 21 samples, causes truncation of the C-terminal autophosphorylation domain encoded by exons 25–28 (Fig. 5a). C-terminal loss is also the most common EGFR fusion reported by TCGA; all of these were also detected by CICERO. In some cases, multiple fusion transcripts leading to EGFR C-terminal truncation can be detected in the same tumor sample suggesting possible clonal heterogeneity [30]. For example, five fusion transcripts causing EGFR C-terminal truncation were predicted by CICERO in sample TCGA-06-2557 (Additional file 1: Figure S4). While one of these five fusions, an in-frame EGFR-SEPT14 [27] fusion with 8 supporting reads, was previously reported by TCGA Research Network; the remaining four fusions, including the predominant out-of-frame EGFR-SDK1 fusion with a total of 357 fusion-positive reads, were not reported. Altogether, 13% of the TCGA samples harbor fusions that can cause C-terminal truncation, a much higher rate than the 4% (7 cases) detected by the TCGA Research Network [27].

Notable examples of non-EGFR fusions not reported by TCGA include three in-frame kinase fusions, i.e., KLHL7-BRAF, CEP85L-ROS1, and TMEM165-PDGFRA (Fig. 5b), and a CCDC127-TERT fusion (Fig. 5c). The three kinase fusions all retain the kinase domain (Fig. 5b). While KLHL7-BRAF has not been reported in GBM previously, it was detected in thyroid papillary carcinoma [31]. CEP85L-ROS1 and TMEM165-PDGFRA were reported by a previous study that defines the landscape of kinase fusions in cancer [32]. The CCDC127-TERT fusion led to activation of TERT expression as the fusion-positive sample (TCGA-06-2564) has the second highest TERT expression of the entire TCGA cohort (Fig. 5c). This fusion was also reported in a previous study that investigated the landscape of cancer-associated transcript fusions [10].

## Discussion

Our motivation for developing CICERO stemmed from a need to detect complex fusions, such as IGH-EPOR, which are highly relevant to targeted therapy but missed by many popular fusion detection tools [7]. The local assembly implemented in CICERO takes advantage of the longer RNA-seq read length (≥ 75 bp) generated by the current NGS sequencing platforms, greatly improving the precision in mapping fusion breakpoints even in highly repetitive regions. Consequently, the most prominent performance improvement of CICERO over existing methods is the ability to detect non-canonical fusions and ITDs from RNA-seq data alone (Fig. 4). Non-canonical fusions detectable by CICERO include chimeric enhancer RNAs which can serve as a surrogate for oncogenic activation via enhancer hijacking (Additional file 1: Figure S3). Since not all enhancer RNAs are polyadenylated [33], the use of total RNA-seq protocol can potentially improve the detectability of enhancer hijacking events by RNA-seq alone.

As with many other fusion detection methods [19], CICERO’s sensitivity is affected by read length, mapping algorithm, and fusion expression level. Short read lengths (e.g., < 75 bp) can result in reduced sensitivity (Additional file 1: Figure S5) likely due to the lower abundance and reduced mappability of RNA-seq reads spanning fusion breakpoints, which are a required input for CICERO. As expected, CICERO performs well for RNA-seq data mapped by algorithms such as STAR [34] which can align reads spanning fusion breakpoints using a soft-clipped mapping signature (Additional file 1: Figure S5), but not for data mapped by algorithms designed to align the full length of RNA-seq reads (e.g., bowtie [35], whose default setting performs global mapping). For lowly expressed gene fusions such as *KIAA1549-BRAF* in low grade glioma, the sensitivity of detection is low for both CICERO as well as the other commonly used fusion detection methods tested here. Alternative strategies such as targeted searches may be needed for recovery of these known driver gene fusions when using RNA-seq for fusion detection in a clinical setting.

Defining the ground truth data set is a major challenge for evaluating the accuracy of fusion detection methods. In the present study, our benchmark analysis design focused on supporting the primary use case of fusion detection in tumor RNA-seq, i.e., the discovery of driver fusions specifically rather than all fusions generally. To this end, we compiled a truth data set representing different types of driver fusions in patient samples from diverse cancer subtypes. All truth data were discovered from a different omics source, i.e., the orthogonally validated structural variations generated by whole-genome sequencing (WGS) of DNA, RT-PCR, or capture sequencing. By contrast, an alternative approach attempting to base the truth data set on the “wisdom of the crowd” by using consensus calls reported by multiple methods runs the risk of missing driver fusions detectable only by the minority vote. For example, our study has shown that in most cases, non-canonical fusions resulting in kinase activation are detectable only by FusionCatcher and CICERO (Fig. 4e, Additional file 2: Table S2). Consequently, measurements of sensitivity in our study may differ from those based on consensus-calling assessments. For example, FusionCatcher appears to be more sensitive than STAR-Fusion in our benchmark analysis, contrary to a recent study that defines truth by consensus methods [19]. When comparing all predicted gene fusions to a truth data set consisting of fusions cross-validated by somatic structural variations in matching WGS data (Methods), the overall false positive rate is high across all prediction programs, even for high-quality predictions from CICERO (78%), Arriba (82%), and STAR-fusion (91%). This suggests that knowledge-based filtering and manual curation are important steps in finalizing somatic gene fusions analyzed by tumor RNA-seq data.

While we used CICERO’s ranking of RNA-seq read-based fusion score in the benchmark analysis (Fig. 4), the heuristic ranking, which is the final output incorporating both the fusion score and a knowledge-based quality grade, does as expected perform better in ranking true driver fusions (Additional file 1: Figure S6; Additional file 2: Table S2). Knowledge-based filtering is also critical for reducing false positives, as we recognized that many highly recurrent chimeric transcripts lack corroborating structural variations (SVs) from DNA WGS. These events may arise from artifacts such as template switching by reverse transcriptase during cDNA preparation [36] or non-canonical RNA splicing such as transcriptional read-through [37]. For example, several recurrent chimeric transcripts are linked to the highly expressed P2RY8 locus. Although one notable fusion transcript, P2RY8-CRLF2, is a bona fide oncogenic fusion caused by a somatic deletion in DNA, others such as P2RY8-AKAP17A and P2RY8-CD99 lack corroborating SVs from WGS even though they can be validated by RT-PCR and Sanger sequencing (Additional file 1: Figure S7). Therefore, we implemented a “blacklist” filter to remove these events. Recurrent ITDs lacking DNA support are also present; one such example is an ITD of *CREBBP* exon 2, detected in three leukemia samples (i.e., SJETV092_D, SJPHALL005_D, SJPML030005_D1) in our benchmark data set.

C-terminal truncation of EGFR is the most prevalent gene fusion discovered in our re-analysis of TCGA GBM RNA-seq. The hotspot breakpoint of the truncation fusions is at the acceptor site of exon 25 (Fig. 5a), the same as the recently reported EGFR–RAD51 fusion in lung cancer [38], which causes the loss of exons 25–28 encoding the autophosphorylation (AP) domain. Loss of the AP domain by deletion or gene fusion has been reported to be transforming and targetable in GBM and lung cancer [4, 38]. Therefore, CICERO’s improved sensitivity in detecting these fusions can potentially expand the eligibility for treatment with EGFR inhibitors in cancer patients.

Our initial goal for implementing a Cloud-based CICERO pipeline was to broaden the accessibility of this complex workflow by making it accessible via a graphical point-and-click interface. This, coupled with the dynamic visualization features in FusionEditor, allows scientists with no formal training in bioinformatics to perform gene fusion detection followed by expert curation using their biological domain knowledge. The Cloud implementation was renamed “Rapid RNA-seq” on St. Jude Cloud (https://platform.stjude.cloud/tools/rapid_rna-seq) after we recognized that the cloud infrastructure is well suited for the scaling up required for genome-wide mapping of each candidate fusion contig. The Rapid RNA-seq platform is able to complete RNA-seq mapping and fusion detection within 2–5 h (~ 100 million reads per RNA-seq sample) even for tumor genomes that underwent massive rearrangements such as chromothripsis. Consequently, Rapid RNA-seq has become our preferred platform for carrying out time-critical fusion detection for our clinical service at St. Jude Children’s Research Hospital. A notable use case of Rapid RNA-seq is to determine the status of kinase fusion for leukemia and lymphoma samples for patients enrolled in the St. Jude Total Therapy Study 17 protocol (https://www.stjude.org/research/clinical-trials/tot17-leukemia-lymphoma.html).

CICERO has been used to analyze more than 2000 RNA-seq samples generated by the two largest pediatric cancer genomics initiatives: the St. Jude/Washington University Pediatric Cancer Genome Project (PCGP) and the Therapeutically Applicable Research to Generate Effective Treatments (TARGET) project. Notable findings to date include C11orf95-RELA fusions that define supratentorial ependymoma [20], targetable kinase fusions in pediatric acute lymphoblastic leukemia (ALL) associated with poor outcome [21], NTRK fusions in pediatric high-grade glioma leading to new therapeutic options [39], and targetable MAP3K8 fusion in pediatric melanoma [5]. We anticipate that the public availability of CICERO will also lead to improved fusion analysis for adult cancer RNA-seq data, as demonstrated through our re-analysis of TCGA GBM in this study and our recent discovery of MAP3K8 C-terminal truncation fusion in 2% of TCGA melanoma samples [5].

## Conclusions

CICERO enables detection of diverse types of gene fusions in RNA-seq, greatly improving our ability to discover non-canonical fusions and ITDs which are overlooked by existing fusion detection methods. A cloud-based implementation, named Rapid RNA-seq, not only enables broad accessibility of CICERO via a graphical point-and-click interface, it also ensures rapid turn-around time by leveraging the Cloud computing infrastructure, supporting time-critical services. The cloud pipeline is also accessible via a command-line workflow for batch job submission. CICERO is freely available for research use at https://github.com/stjude/Cicero. A computational cloud implementation of CICERO is available at https://platform.stjude.cloud/tools/rapid_rna-seq.

## Methods

### Fusion detection by breakpoint analysis

The input for the fusion detection process is a BAM file generated by mapping of paired-end RNA-seq data using algorithms such as STAR [34] followed by removal of duplicate reads with Picard [40]. Candidate fusions are discovered by breakpoint analysis involving the following three steps: (1) identification of candidate fusion breakpoints marked by soft-clipped (SC) reads (SCreads), (2) assembly of the fusion contig, and (3) mapping of the fusion contig for discovery of the partner locus breakpoint.

Candidate fusion breakpoints are initially identified by the presence of SCreads, i.e., RNA-seq reads that contain soft-clipped subsequences in their mapping to the reference human genome. To account for mapping ambiguity, SCreads within 3 base pairs of one another are considered a SC cluster, and the position of the cluster is denoted by the position of the SCreads with the longest soft-clipped subsequence. As a SC signature requires at least 20-bp read length, we adjusted the RNA-seq read-count based expression, represented by the variable adjusted_gene_exp, by penalizing short read length with a parameter *w*, as “(read_length-20)/100” to reflect the fact that reads with < 20 bp will generally not be soft-clipped by the aligner:

$$ \mathrm{adjusted}\_\mathrm{gene}\_\exp =w\times \frac{\mathrm{read}\_\mathrm{cnt}}{\mathrm{mRNA}\_\mathrm{length}} $$

We use SC_cnt to quantify the total number of SCreads for each SC cluster. An SC cluster is required to meet the following criteria to be considered a candidate fusion breakpoint: (i) SC_cnt ≥ 2 for a genic site or SC_cnt ≥ 5 for an intergenic site; (ii) adjusted_gene_exp ≥ 0.01 for a genic site, to avoid artifacts with very low expression; (iii) the site does not match the highly paralogous regions with excessive mapping artifacts (e.g., ribosome, hemoglobin); and (iv) SCreads transcriptional allelic fraction (SC_TAF), defined as SC_cnt/total mapped reads, exceeds 0.05.

For each candidate breakpoint, denoted as bp1, SCreads and their paired mates, along with discordantly mapped read pairs present in the region, are assembled into contigs using CAP3 (with parameters −o 25 −z 2 −h 60 −y 10) [41]. To reduce mapping ambiguity, discordantly mapped read pairs are also included in the assembly but only when the mate of the mapped read is projected to have the potential to extend past the fusion junction as illustrated in Additional file 1: Figure S8. Read pairs with one mapped and one unmapped read are also considered discordantly mapped read pairs in order to account for mapping failures attributable to non-templated insertions [7].

The assembled contig for bp1 is mapped to the reference human genome using BLAT (−minScore 25 and outputting the top 3 best hits) to determine the validity of candidate fusions and to find the partner breakpoint, denoted bp2. Two rounds of BLAT search are performed. The first round uses the entire contig, and if the full-length contig is mapped to a non-bp1 genomic location, bp1 is discarded as an artifact of paralogous mapping. Otherwise, the portion of the contig not mapped to bp1 is considered to represent the sequence at bp2, denoted as s2_,_ and will be used as the query for the second BLAT search. If s2 is mapped to multiple locations, bp2 is prioritized for regions with proximity to bp1 (i.e., within the same gene or within 100 kb of bp1), a conservative approach that prioritizes potential local events or library artifacts over gross genomic alterations. Multiple mapping of s2 is not penalized, enabling discovery of fusions in highly repetitive regions. A contig with both bp1 and bp2 located in highly paralogous regions (e.g., ribosomal RNA, immunoglobin, T cell receptor, and HLA loci; Additional file 2: Table S6) or matching the structural variations resulting from V(D) J recombination is considered false positive and is not subjected to further evaluation. BLAT search can become a computational bottleneck for tumor genomes that have undergone massively catastrophic rearrangements known as chromothripsis [42], which motivated the deployment of CICERO on the St. Jude Cloud platform where each CICERO run launches its own private BLAT server on the same host running the CICERO code.

### Fusion detection by analysis of splice junction reads

Some fusion transcripts caused by a deletion may lack soft-clipped reads, as reads spanning a fusion junction may be mapped as splice junctions by the RNA mapper (e.g., < 590 Kb for splice junction supported by ≥ 4 reads in the default setting of for STAR 2.7). One such example can be seen in fusion junction reads for P2RY8-CRLF2, a common oncogenic fusion in leukemia [6] caused by deletions spanning ~ 300Kb (Additional file 1: Figure S2). Therefore, we implemented a complementary “rescue” process to enable fusion detection from novel splice junctions absent from the reference gene model with the following criteria: (i) the splice junction reads span ≥ 10 kb and encompass ≥ 2 genes and (ii) the transcript allelic fraction of junction reads exceeds 0.01.

### Fusion reading frame annotation

If coding exons from two genes are joined by a fusion contig, CICERO performs automated frame-checking by translating the fusion contig and matching the protein sequence to each fusion partner (Additional file 1: Figure S9). We use UCSC refFlat mRNA genome mappings and associated protein products from refSeq. De novo translations of the mappings are performed to verify that each produces the refSeq protein product with up to 4% mismatch permitted. The process generates three alternative protein coding frames from the fusion contig and then attempts to anchor the two partner genes, gene A and gene B, to each.

The anchoring process begins with gene B as this provides a better model for the transcription activation events such as promoter swapping: the code identifies all transcripts overlapping the breakpoint and then searches each transcript’s genomic mapping from the breakpoint downstream until encountering coding sequence (Additional file 1: Figure S9b). A sample of downstream amino acid sequence is then extracted and searched for in the three coding frames to determine which is in-frame with gene B. The default search tuple size is 10 amino acids, which may be increased if necessary to find a unique match, or decreased if the event falls near the end of the transcript. Synthetic codons are generated into the 5′ UTR to aid anchoring in these regions, as the fusion contig may not provide coverage of the coding sequence in these situations (Additional file 1: Figure S9c).

If the tuple search method is unable to identify a matching frame (due to, e.g., minor sequence variation) BLAT is used as an alternative anchoring method, similarly requiring a minimum 10-AA match (a “−minScore” value of 20 is used for increased sensitivity). When BLAT is used, the fusion contig is masked to the gene B portion to avoid ambiguous anchoring in single-gene internal events. Once the correct coding frame for gene B has been identified, a similar search procedure is followed for gene A, this time seeking upstream into that transcript, to determine whether this frame is compatible with gene A’s coding.

### Evidence-based ranking of fusion candidates

To better distinguish bona fide gene fusions from RNA-seq artifacts, we implemented an evidence-based ranking process in CICERO to prioritize fusion candidates during a manual review. The ranking is based on a number of factors: fusion allele frequency, matching length, repetitive mapping, and frame-check results with a quality status determined by matches to known fusion events or artifacts.

The following variables are defined to calculate a fusion score:

We define the weight of fusion transcript allele frequency (TAF) of soft-clipped reads or splice junction reads (w_TAF) at a candidate locus as follows:

$$ \mathrm{w}\_\mathrm{TAF}=\left\{\begin{array}{ll}1& \mathrm{TAF}\ge 0.01\\ {}{e}^{-\frac{0.01}{\mathrm{TAF}}}& \mathrm{TAF}<0.01\end{array}\right. $$

The weight of matched contig length (w_Match) at a position:

$$ \mathrm{w}\_\mathrm{Match}=\Big\{{\displaystyle \begin{array}{c}1\kern0.9cm \mathrm{match}\_\mathrm{length}\ge \kern0.1cm 0.5\times \mathrm{read}\_\mathrm{length}\\ {}{e}^{\kern0.1cm \frac{\mathrm{match}\_\mathrm{length}-0.5\times \mathrm{read}\_\mathrm{length}}{2}}\kern0.3cm \mathrm{match}\_\mathrm{length}<0.5\times \mathrm{read}\_\mathrm{length}\end{array}}\operatorname{} $$

match_length refers to the matching portion of the contig length at the position.

The score of a fusion at each partner breakpoint (score_*p*):

$$ \mathrm{score}\_p=\mathrm{w}\_\mathrm{TAF}\times \mathrm{w}\_\mathrm{Match}\times \mathrm{area}\times \left(1-\mathrm{repeat}(p)\right), $$

where $\mathrm{repeat}(p)=1-\frac{\mathrm{matches}(p)}{\sum_i\mathrm{matches}\left({p}_i\right)}$ is a repeat score (range 0–1) of the contig mapping and matches(*p*) is the matched length of the blat hits; *p*_*i*_ is all the possible blat hits with > 90% matched identity to a fusion contig. A repeat value of 0 represents unique mapping. “area” represents the coverage of fusion junction reads as a sum of the length of all subsequences that can be mapped to the fusion contig.

The fusion score combines the score from the two partners, bp1 and bp2, as follows:

$$ \mathrm{score}=0.5\times \left(\mathrm{score}\_p\left(\mathrm{bp}1\right)+\mathrm{score}\_p\left(\mathrm{bp}2\right)\right)\times \mathrm{ort}\times \mathrm{frame} $$

“Ort” is set to 2 if the orientation of the fusion is consistent with the transcription orientation of the two partner genes; otherwise, it is set to 1. Frame is set to 2 and 1 for in-frame and out-of-frame fusions, respectively.

Predicted fusions with score ≥ 1, the repeat score of bp1 and bp2 < 0.7, and TAF at bp1 and bp2 ≥ 0.01 are retained as candidate fusions which are subsequently categorized as high quality (HQ) if they match known gene fusions or ITDs, read-through (RT), or low quality (LQ) for non-read-through novel events. The final ranking proceeds in the order of HQ, LQ, RT, and within each category individual fusions are ranked by fusion score. The known fusion gene list was compiled from COSMIC [43], ProteinPaint [23], and the Mitelman database (https://cgap.nci.nih.gov/Chromosomes/Mitelman) and genes with known ITD (i.e., FGFR1, FLT3, PDGFRA, NOTCH1, EGFR, PIK3R1, BRAF, BCOR, and MYC) were based on literature searches.

### RNA-seq data sets

The benchmark data set was comprised of 170 RNA-seq with 100 bp read length. mRNA-seq and total RNA-seq protocol were used to profile 134 [3, 21] and 36 tumor samples [15], respectively; the details are summarized in Additional file 2: Table S1**.**

The TCGA GBM samples were downloaded from https://tcga-data.nci.nih.gov/docs/publications/gbm_2013/ [27], which contains 167 samples profiled by mRNA-seq with 75 bp read length; the details are summarized in Additional file 2: Table S3.

To compare the fusion transcript detected by CICERO with those reported by TCGA Research Network, we used data from Table S4 by Brennan et al. As only gene names but not genomic coordinates were listed in Table S4, we considered a fusion detected by both TCGA report and CICERO if its two partner genes were matched.

### Public fusion detection tools used for benchmark test

We compared the detectability and ranking of CICERO with the following four widely adopted RNA-Seq fusion detection tools: deFuse [16], ChimeraScan [17], Fusioncatcher [18], STAR-Fusion [19], and Arriba [24]. For deFuse (version 0.6.2), we used the “probability” score for ranking; for ChimeraScan (version 0.4.5), we used “score”; for Fusioncatcher (version 0.99.7d), we used “Spanning_unique_reads”; and for STAR-Fusion (version 1.6.0) and Arriba, the ranking was based on the listed order of the predicted fusions.

### Assessing false positive rates in CICERO and other fusion prediction algorithms

The truth data set used for assessing the false positive (FP) rate of predicted RNA-seq fusions is comprised of fusions that can be validated by somatic DNA structural variations computed from paired tumor-normal WGS data. Among the 170 benchmark samples, 80 have matched tumor-normal whole-genome sequencing data, so we limited our FP analysis to this subset.

For each of these 80 cases, we used curated somatic SVs analyzed by our CREST algorithm [44] as well as putative somatic SVs computed by two recently published methods, Manta [45] and SvABA [46], using the default parameters. A predicted RNA-seq gene fusion is considered valid if both breakpoints are located within 100 Kb of DNA somatic SV breakpoints computed by any of these three WGS SV methods. The 100 Kb interval allows for flexibility in mapping a site on a spliced RNA to its matching DNA region. Using this approach, we were able to verify all except for 6 subclonal RNA-fusions of the 84 driver fusions identified in the 80 cases, indicating a high sensitivity (93%) of this approach in validating gene fusions caused by somatic SVs.

We performed false positive rate analysis on high-quality CICERO fusions which contain all the detected driver fusions presented in the 80 samples, and all CICERO fusions which include both high- and low-quality predictions. Rearrangements within immunoglobulin (e.g., IGH) or T cell receptor (TCR) loci were filtered out as these events occurred during normal B cell or T cell development stage. Fusion transcripts resulting from the same DNA structural variation are scored individually; e.g., the 375 true positive fusions predicted by Arriba were considered as 375 events even though they were supported by 185 unique DNA structure variations. We compared the FPs of high-quality predictions with those of STAR-fusion and Arriba as these methods have a comparable total number of predicted gene fusions. Based on a previous report [19] as well as our own experience, we included all predictions by Arriba in this analysis as excluding low-confidence (Additional file 1: Figure S10) or read-through events impairs overall sensitivity without providing a major improvement in accuracy. For the set of all CICERO predictions, we compared FPs with the other three algorithms, i.e., deFuse, ChimeraScan, and FusionCatcher.

### CICERO on St. Jude Cloud

An end-to-end pipeline deployable through a graphical point-and-click interface is available on St. Jude Cloud (https://platform.stjude.cloud/tools/rapid_rna-seq). The cloud pipeline can accept either unaligned reads in fastq format or a BAM file generated by STAR mapping [34]. A major advantage of the Cloud pipeline is to effectively manage the burst of computing capacity required for running BLAT search for samples that have massive numbers of gene fusions caused by massive genomic rearrangement events such as chromothripsis. In addition, the Cloud pipeline also performs low-stringency fuzzy matching of every read in the BAM file in order to rescue fusion junction reads regardless of whether they have been aligned or not in a module named Fuzzion. The Fuzzion algorithm is able to rescue low-expressed gene fusions such as KIAA1549-BRAF and KMT2A-MLLT3 [15] that may fall below CICERO’s limit of detection; it is able to recover even a single low-quality read that potentially supports a known fusion gene junction. The Fuzzion output is a simple text file with read IDs and 20-mer sequences supporting a particular fusion gene junction.

## Supplementary information

**Additional file 1.** Additional figures. This file presents all supplementary figures referenced in the main text.

**Additional file 2.** Additional tables. This file contains information about benchmark samples (tab 1), performance evaluation of benchmark samples by different algorithms (tab 2), sample information about TCGA GBM cohort (tab 3), cancer gene fusions identified in TCGA GBM cohort (tab 4 & 5) and reference files used in CICERO analysis (tab 6).

**Additional file 3.** Additional tables. This file shows the results of false positive rate of different fusion detection methods based on the analysis of 80 cases that have both WGS and RNA-seq.

**Additional file 4.** Review history.

## Data Availability

Links to CICERO source code and benchmark data are maintained at https://pecan.stjude.cloud/permalink/cicero. The CICERO source code is available at https://github.com/stjude/Cicero [47] and https://zenodo.org/record/3817590. DOI: 10.5281/zenodo.3817590 [48]. CICERO is licensed under a modified version of the Apache License (Version 2.0) for free academic research use. A cloud implementation of CICERO is available at https://platform.stjude.cloud/tools/rapid_rna-seq. FusionEditor is available at https://proteinpaint.stjude.org/FusionEditor/. The benchmark dataset is from EGAS00001000255 EGAS00001002217 EGAS00001000654 EGAS00001000192 EGAS00001000254 EGAS00001000256 EGAS00001000349 EGAS00001000447 EGAS00001000449 EGAS00001003266. The TCGA GBM samples were from https://tcga-data.nci.nih.gov/docs/publications/gbm_2013/. Most of the benchmark dataset is also available in St. Jude Cloud at https://platform.stjude.cloud/data/cohorts?dataset_accession=SJC-DS-1010.
