# Supplementary material for: CICERO: a versatile method for detecting complex and diverse driver fusions using cancer RNA sequencing data
Source: Genome Biol. 2020 May 28;21:126. doi: 10.1186/s13059-020-02043-x (PMC7325161; doi:10.1186/s13059-020-02043-x)
Supplement: Supplementary file 1 — Additional file 1. Additional figures. This file presents all supplementary figures referenced in the main text. [file 13059_2020_2043_MOESM1_ESM.docx]

**Figure S1** Visualization of predicted fusions in 167 TCGA-GBM RNA-seq in FusionEditor. **a** Summary of predicted fusions of 3 representative samples in FusionEditor. **b** Table view that lists recurrent fusions in the cohort. **c** Search result of TERT related fusions. **d** Graphical view of CCDC127-TERT in-frame fusion. The bottom panels show the expression level (FPKM) of the partner genes in the cohort with the fusion-positive sample highlighted in red. **e** Detailed information on the CCDC127-TERT fusion.


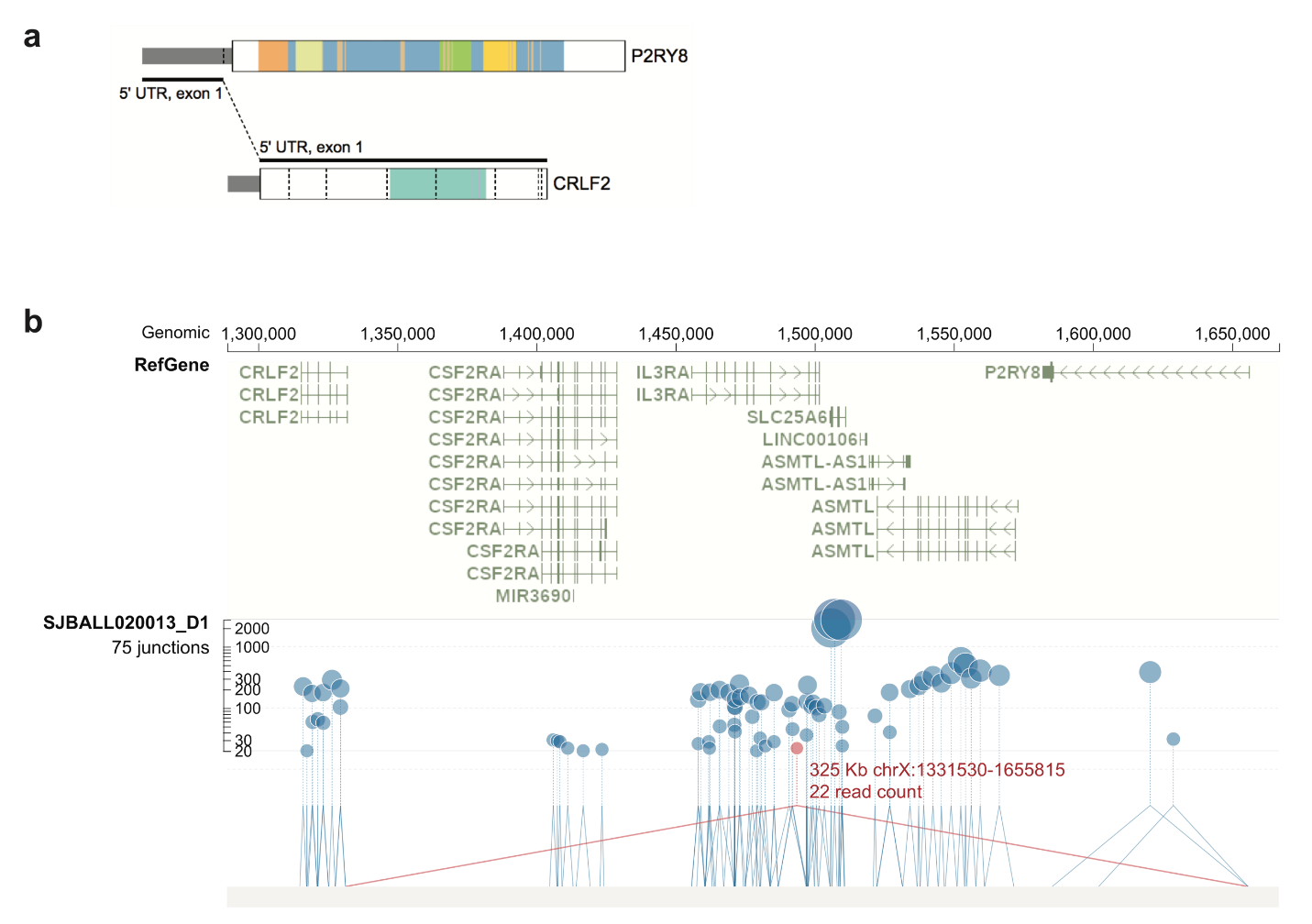


**Figure S2** Novel junction reads rescue P2RY8-CRLF2 fusion in a B-ALL patient (SJBALL020013_D1). **a** FusionEditor visualization of the P2RY8-CRLF2 fusion which causes promoter swapping by merging 5’ UTR of P2RY8 with the first exon of CRLF2. **b** RNA-seq read count of splice site junctions in the 350Kb region compassing P2RY9 and CRLF2 shown by the GenomePaint viewer(<https://genomepaint.stjude.cloud/>) . The circle represents a known and novel splice junction in blue and red, respectively. The y-axis and size of the circle correspond to the junction read count. The novel splice junction representing P2RY8-CRLF2 fusion is highlighted.


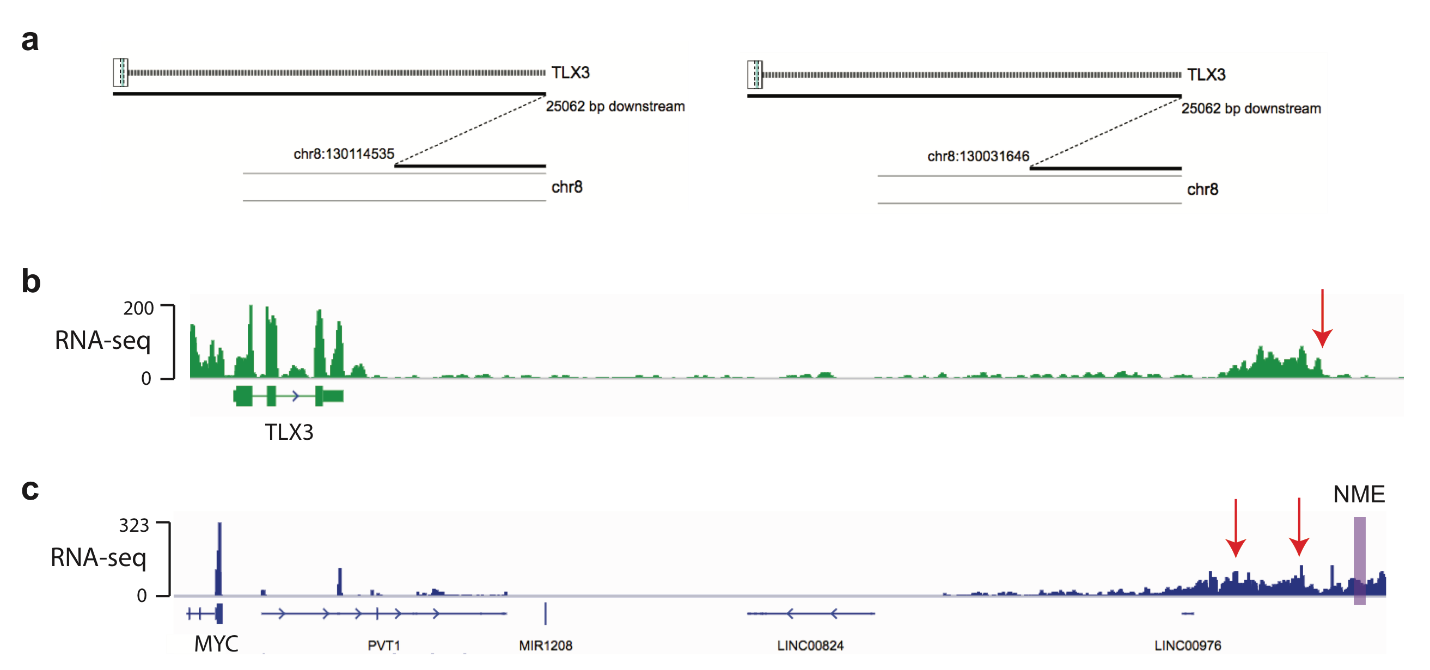


**Figure S3** TLX3 dysregulation by enhancer-hijacking detected as a non-canonical fusion of enhancer RNA. The RNA-seq for this sample (SJTALL030071_D1), generated by total RNA-seq protocol, is available on St. Jude Cloud as part of the Genomes for Kids cohort (<https://platform.stjude.cloud/requests/cohorts>). **a** FusionEditor view of two non-canonical fusions involving TLX3 and chr8 MYC enhancer region predicted by CICERO. **b** RNA-seq coverage around the TLX3 and its enhancer regions. The fusion breakpoint is shown as red arrow. **c** RNA-seq coverage at the MYC locus. The fusion breakpoint is shown as red arrow. The NOTCH-bound MYC enhancer [1] (NME), specific for T-ALL, is highlighted in purple.

**Figure S4** Five distinct fusion transcripts of EGFR C terminal truncation in TCGA-06-2557. Fusion partners, marked by different colors, connect to the same EGFR fusion breakpoint at the splice donor site of exon 24 of EGFR, leading to truncation of exons 25-28. The abundance of each fusion transcript is marked by the number of soft-clipped or discordantly paired end reads supporting the fusion transcript. EGFR-SEPT14, marked with *, is the only fusion transcript reported by the TCGA Research Network [2]. Soft-clipped reads representing distinct fusion transcript are shown as letters in gray using the Bambino viewer [3].

**Figure S5** Comparison of the sensitivity (top panel) and ranking (bottom panel) of the driver fusions detected by CICERO using different mapping methods and RNA-seq read size. Fusion genes are shown in the four categories of highly expressed chimeric transcript, low-expressed chimeric transcript, internal tandem duplication (ITD), and other non-canonical fusions involving intronic or intergenic regions. The first two columns show the results based on our StrongArm [4] mapping pipeline (red color) of 100-bp read size and STAR mapping (green color) of 100-bp read size while the 3^rd^ column (blue color) displays the results by STAR mapping of using RNA-seq reads trimmed to a short 50-bp length.

**Figure S6** Comparison of two ranking methods of CICERO. The ranking based on fusion score, denoted as CICERO_raw, is based on RNA-seq read count and used in Fig. 4 to ensure fairness of comparison. The heuristic ranking based on both the fusion score and a knowledge-based quality grade is denoted as CICERO and it is the final ranking output by the CICERO package. The ranking of other five methods (i.e. ChimeraScan, deFuse, FusionCatcher, STAR-Fusion and Arriba) are also displayed.


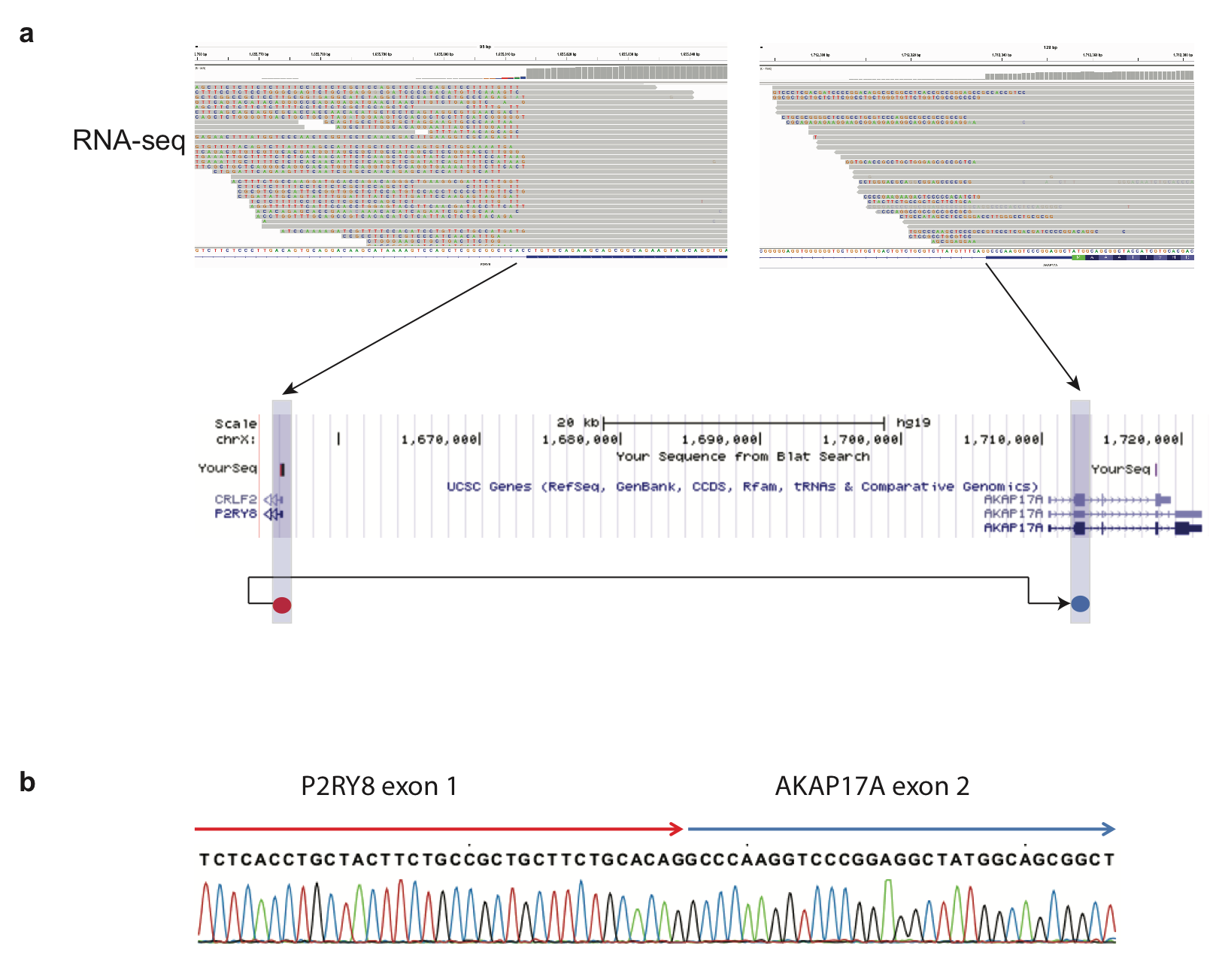


**Figure S7** An example of an expressed chimeric transcript (P2RY8-AKAP17A in sample SJBALL020984_D1) in RNA-seq but lacking structural variation support at the DNA level. This event is likely caused by a transcription read-through event. **a** Soft-clip reads at both chimeric fusion breakpoints from RNA-seq. Mapping of the fusion contig shown at the bottom. **b** Sanger sequencing confirming the chimeric transcript.

**Figure S8** Selection of discordant and soft-clipped read pairs for contig assembly in CICERO. For discordant pairs near the breakpoint, only the pairs that have the potential to extend the fusion junction are included as indicated by the green check mark.

**Figure S9** Fusion frame-checking process. **a** Process overview: combinations of transcript pairs for both genes A and B first have their genomic mappings validated, then frame status is evaluated by comparing the 3 possible coding frames derived from the CICERO contig sequence with the expected gene protein sequence at each gene’s respective genomic breakpoint. **b** In-frame example: 3 possible protein coding frames are generated from the genomic fusion contig. The sequence in coding frame 3 matches the protein sequence for genes A and B mapped at their respective breakpoints. **c** Special handling for gene B 5’ UTR: in some cases the fusion contig sequence may overlap the 5’ UTR but not extend into the coding sequence. Synthetic codons are generated based on the 5’ UTR sequence which are used to identify which candidate coding frame is in-frame with gene B’s downstream coding sequence.

**Figure S10** Comparison of the sensitivity (top panel) and ranking (bottom panel) of the driver fusions detected by HQ versus all Arriba prediction. Fusion genes are shown in the four categories of highly expressed chimeric transcript, low-expressed chimeric transcript, internal tandem duplication (ITD), and other non-canonical fusions involving intronic or intergenic regions.

**References**

1. Herranz D, Ambesi-Impiombato A, Palomero T, Schnell SA, Belver L, Wendorff AA, Xu L, Castillo-Martin M, Llobet-Navas D, Cordon-Cardo C, et al: **A NOTCH1-driven MYC enhancer promotes T cell development, transformation and acute lymphoblastic leukemia.** *Nat Med* 2014, **20:**1130-1137.

2. Brennan CW, Verhaak RG, McKenna A, Campos B, Noushmehr H, Salama SR, Zheng S, Chakravarty D, Sanborn JZ, Berman SH, et al: **The somatic genomic landscape of glioblastoma.** *Cell* 2013, **155:**462-477.

3. Edmonson MN, Zhang J, Yan C, Finney RP, Meerzaman DM, Buetow KH: **Bambino: a variant detector and alignment viewer for next-generation sequencing data in the SAM/BAM format.** *Bioinformatics* 2011, **27:**865-866.

4. Rusch M, Nakitandwe J, Shurtleff S, Newman S, Zhang Z, Edmonson MN, Parker M, Jiao Y, Ma X, Liu Y, et al: **Clinical cancer genomic profiling by three-platform sequencing of whole genome, whole exome and transcriptome.** *Nat Commun* 2018, **9:**3962.
