# Supplementary material for: CICERO: a versatile method for detecting complex and diverse driver fusions using cancer RNA sequencing data
Source: Genome Biol. 2020 May 28;21:126. doi: 10.1186/s13059-020-02043-x (PMC7325161; doi:10.1186/s13059-020-02043-x)
Supplement: Supplementary file 4 — Additional file 4. Review history. [file 13059_2020_2043_MOESM4_ESM.docx]

Review History

**First round of review**

**Reviewer 1**

**Were you able to assess all statistics in the manuscript, including the appropriateness of statistical tests used?**

Yes, and I have assessed the statistics in my report.

**Were you able to directly test the methods?**

No

**Comments to author:**

Predicting gene fusions from transcriptome data is important for cancer diagnosis and therapy. This study presents a novel software package, CICERO for gene fusion. The authors developed a new algorithm, built an online portal, and applied the tool to several cancer genome projects. Compared to existing methods, the performance is substantially improved and have many advantages. The overall design is quite straightforward and the results are solid. Both the software package and the cloud portal prove will be important resources for the biomedical research community. I have a few points to improve the study.

1.     A major challenge for most gene-fusion algorithms is not about having fewer true positives, but  too many false positives. The authors showed that CICERO was able to recapture most of the true positives, but it is likely CICERO may also introduce some or many false positives. I'd suggest that the authors provide some summaries and discussions for total prediction results, instead of focusing on so called "ground truth positives".

2.     Alignment tools, sequence length, insert size and gene expression are several factors that could affect the performance of those tools. Considering that those factors were not assessed in the benchmark, the authors should at least provide some discussion about these factors.

3.     Error bars should be added to c, d, e, f in Figure 4. This could be done by bootstrapping analysis. So it is easy to know whether the difference is meaningful.

4.     In Abstract "CICERO outperforms existing methods". Since quite a number of methods were not included in the assessment, it should be "CICERO outperforms commonly used methods"

5.     "Cancer transcriptome sequencing (RNA-seq) data" in the title is a little awkward. It could be "cancer RNA-sequencing data"

Recommended, but not required:
1.     Simulating RNA-seq reads with predefined fusions could serve as a good benchmark data complementary with real cancer data. It could also help generate more convincing benchmark statistics for the five algorithms comparison.

**Reviewer 2**

**Were you able to assess all statistics in the manuscript, including the appropriateness of statistical tests used?**

Yes, and I have assessed the statistics in my report.

**Were you able to directly test the methods?**

No

**Comments to author:**

This paper introduces a new method for gene fusion detection from RNA-seq data named CICERO. CICERO is a local assembly-based algorithm that integrates RNA-seq read support, identifies the candidate breakpoints, assembles the reads, maps the contigs and ranks the detected gene fusions after a fusion annotation process. This method is able to discover not only the canonical exon-to-exon chimeric transcripts, but also non-canonical fusions including internal tandem duplications, intronic and intergenic regions, etc.
To evaluate the performance of CICERO, the authors have used a dataset of 184 driver gene fusions discovered in 170 samples of various types of cancer. They have compared the performance of CICERO with four other existing tools namely ChimeraScan, deFuse, Fusion-Catcher, and STAR-Fusion. CICERO outperforms the other methods in terms of sensitivity and the ranking of the candidate fusions. They have also run CICERO on RNA-seq data from The Cancer Genome Atlas Gliobastoma Multiforme which includes 167 samples and resulted in detection of novel gene fusions.

The manuscript is well-written and the method is sound. I did not download the software or run it, so can't comment on that.

I have two main comments:
1- Although "high false positives" is one of the motivations to develop this novel pipeline, only sensitivity and ranking of the driver fusions of the methods have been mentioned in the evaluation part of the paper. I believe the authors should provide tables/figures showing the specificity of the tools used for benchmarking.

2- As mentioned in this paper, a recent article titled "Accuracy assessment of fusion transcript detection via read-mapping and de novo fusion transcript assembly-based method" (https://genomebiology.biomedcentral.com/track/pdf/10.1186/s13059-019-1842-9) has assessed 23 methods for detection of gene fusions. From that study, STAR-Fusion, Arriba, and STAR-SEQR are reported as the most accurate and fastest for fusion detection on cancer transcriptome. The set of methods used here for benchmarking only include STAR-Fusion from the aforementioned assessment study. Given the findings in the benchmarking study, I believe the authors should also run Arriba and STAR-SEQR.

**Authors Response**

**Point-by-point responses to the reviewers’ comments:**

Reviewer #1: Predicting gene fusions from transcriptome data is important for cancer diagnosis and therapy. This study presents a novel software package, CICERO for gene fusion. The authors developed a new algorithm, built an online portal, and applied the tool to several cancer genome projects. Compared to existing methods, the performance is substantially improved and have many advantages. The overall design is quite straightforward and the results are solid. Both the software package and the cloud portal prove will be important resources for the biomedical research community. I have a few points to improve the study.

*[Response] We thank the reviewer for recognizing the advantages of using CICERO for gene fusion detection and for suggesting useful additional analyses.*

1.      A major challenge for most gene-fusion algorithms is not about having fewer true positives, but too many false positives. The authors showed that CICERO was able to recapture most of the true positives, but it is likely CICERO may also introduce some or many false positives. I'd suggest that the authors provide some summaries and discussions for total prediction results, instead of focusing on so called "ground truth positives".

*[Response] We thank the reviewer for the suggestion of evaluating the false positives (FP) amongst all the predicted fusions. A major challenge in assessing FP is the need to establish a truth data set. As discovery of somatically-acquired gene fusions is the main application for RNA-seq fusion detection, we decided to use RNA-seq fusions validated by somatic structural variations computed from paired tumor-normal DNA WGS as the truth data set for this analysis. This allows us to use DNA WGS as an orthogonal validation to determine the accuracy of fusions generated from RNA-seq. Among the 170 samples used for our benchmark analysis, 80 have matched tumor-normal whole-genome sequencing data, so we limited our FP analysis to this subset of the data. To ensure the sensitivity of somatic WGS SV calling, we took the union of somatic DNA SVs predicted by three algorithms (CREST, Manta and SvABA) for cross-validating predicted RNA-seq fusions. To account for the difference between a spliced RNA and its DNA coordinates, a predicted RNA-seq gene fusion is considered valid if both breakpoints are located within 100Kb of DNA somatic SV breakpoints computed by any of these three methods. By using this approach, we are able to generate the true positives for evaluating FP rate without relying on simulation or the “wisdom of crowd”.*

*In the revision, we incorporated this new analysis by 1) adding a new section “Assessing false positive rates in CICERO and other fusion prediction algorithms” in Methods; 2) revising the Results section “Comparison of CICERO with other methods of detecting diverse driver gene fusions” by changing the subtitle to “Comparison of CICERO with other methods of detecting somatic gene fusions” as the analysis has been expanded to all predicted gene fusions based on the review’s feedback. A new paragraph along with a new panel of Figure 4, Figure 4d, has been added to summarize the false positive rate of high-quality predictions as well all predicted fusions (shown below). This analysis also includes an additional method Arriba based on the feedback by Reviewer 2; 3) revising the title of the manuscript from “CICERO: an accurate method” to “CICERO: a versatile method” to better capture comprehensiveness of the analysis provided by CICERO; and 4) adding a discussion of high false positive rates to the third paragraph of the Discussion as follows:*

*“When comparing all predicted gene fusions to a truth dataset consisting of fusions cross-validated by somatic structural variations in matching WGS data (Methods), the overall false positive rate is high across all prediction programs, even for high-quality predictions from CICERO (78%), Arriba (82%), and STAR-fusion (91%). This suggests that knowledge-based filtering and manual curation are important steps in finalizing somatic gene fusions analyzed by tumor RNA-seq data.”*

*Figure 4. d) True positives (dark blue) and false positives (light blue) of predicted somatic fusions identified by CICERO and other fusion detection programs. The exact number of events is marked as (true positive/total prediction) under the name of each method. CICERO’s high-quality predictions are compared to those of STAR-fusion and Arriba (left panel) while all CICERO predictions are compared to those of FusionCatcher, deFuse, and ChimeraScan (right panel).*

2. Alignment tools, sequence length, insert size and gene expression are several factors that could affect the performance of those tools. Considering that those factors were not assessed in the benchmark, the authors should at least provide some discussion about these factors.

*[Response] We thank the reviewer for this suggestion as factors affecting the sensitivity of fusion detection have also been investigated previously by Haas et al (Genome Biol. 2019 Oct 21;20(1):213). We re-analyzed the benchmark data set by trimming the RNA-seq read to 50bp and by using an alternative mapping method. These results are presented in a new Supplementary Figure (Additional file 1: Figure S5 shown below) while reduced sensitivity in detecting lowly expressed gene fusion has already been shown in Figure 4. We did not discuss the effect of varying library insert size in RNA-seq because the varying library insert size is more relevant to SV analysis of whole-genome DNA sequencing. For RNA sequencing, an insert size of 120-225bp is a standard practice due to the need to capture the majority of expressed transcripts. Samples with a peak insert size distribution of < 120bp generally indicate a significant issue with the initial RNA quality and would not pass our quality check (QC) for RNA-seq data. These samples are expected to have reduced library diversity and potentially affect the ability for fusion detection.*

*In the revised manuscript we have included a new section in Discussion that comments on the effect of mapping algorithms, sequence length and gene expression on fusion detection as follows:*

*“As with many other fusion detection methods [19], CICERO’s sensitivity is affected by read length, mapping algorithm, and fusion expression level. Short read lengths (e.g. <75bp) can result in reduced sensitivity (Additional file 1: Figure S5) likely due to the lower abundance and reduced mappability of RNA-seq reads spanning fusion breakpoints, which are a required input for CICERO. As expected, CICERO performs well for RNA-seq data mapped by algorithms such as STAR [34] which can align reads spanning fusion breakpoints using a soft-clipped mapping signature (Additional file 1: Figure S5), but not for data mapped by algorithms designed to align the full length of RNA-seq reads (e.g. bowtie [35], whose default setting performs global mapping). For lowly expressed gene fusions such as KIAA1549-BRAF in low grade glioma, the sensitivity of detection is low for both CICERO as well as the other commonly-used fusion detection methods tested here. Alternative strategies such as targeted searches may be needed for recovery of these known driver gene fusions when using RNA-seq for fusion detection in a clinical setting.”*

*Figure S5 Comparison of the sensitivity (top panel) and ranking (bottom panel) of the driver fusions detected by CICERO using different mapping methods and RNA-seq read size. Fusion genes are shown in the four categories of highly expressed chimeric transcript, low-expressed chimeric transcript, internal tandem duplication (ITD), and other non-canonical fusions involving intronic or intergenic regions. The first two columns show the results based on our StrongArm [4] mapping pipeline (red color) of 100-bp read size and STAR mapping (green color) of 100-bp read size while the 3rd column (blue color) displays the results by STAR mapping of using RNA-seq reads trimmed to a short 50-bp length.*

3.      Error bars should be added to c, d, e, f in Figure 4. This could be done by bootstrapping analysis. So it is easy to know whether the difference is meaningful.

*[Response] We thank the reviewer for this suggestion. For each group of gene fusions, we performed the bootstrapping analysis, calculating sensitivity and repeated the analysis 100 times. The standard deviation of the 100 iterations is shown as the error bar in the revised Figure 4c which now combines the data in the original c, d, e and f (shown below). The figure also included an additional method Arriba based on reviewer 2’s suggestion. We included the following text in the revised figure legend: “Error bars for fusion detection sensitivity (top panel) were calculated by bootstrapping with 100 iterations.”*

*Figure 4. c) Comparison of the sensitivity (top panel) and ranking of the driver fusions amongst all predicted fusions (bottom panel) by CICERO and five other methods (ChimeraScan, deFuse, FusionCatcher, STAR-Fusion and Arriba) in the four categories of driver fusion. The ranking by CICERO, labeled CICERO_raw, is based on fusion score alone without incorporating matches to known fusion status. Error bars representing standard deviation of detection sensitivity at the top panel were calculated by bootstrapping of samples with 100 iterations*

4.      In Abstract "CICERO outperforms existing methods". Since quite a number of methods were not included in the assessment, it should be "CICERO outperforms commonly used methods"

*[Response] We thank the reviewer for this great suggestion and have revised the abstract by changing “existing methods” to “commonly used methods”.*

5.      "Cancer transcriptome sequencing (RNA-seq) data" in the title is a little awkward. It could be "cancer RNA-sequencing data"

*[Response] We thank the reviewer for this suggestion and have modified the title as follows: “CICERO: a versatile method for detecting complex and diverse driver fusions using cancer RNA sequencing data”.*

Recommended, but not required:
1.      Simulating RNA-seq reads with predefined fusions could serve as a good benchmark data complementary with real cancer data. It could also help generate more convincing benchmark statistics for the five algorithms comparison.

*[Response] We thank the reviewer for suggesting an analysis using simulated data. After carefully evaluating this suggestion, we decided not to perform simulation as we are concerned that a simple simulation may not be appropriate for measuring the performance of CICERO and other methods in detecting gene fusions using real clinical samples. Our past experience has shown that the accuracy of fusion detection using RNA-seq data is affected by many confounding factors, some of which are difficult to model appropriately, e.g. as variations in fusion gene expression level, mappability of break points of the partner genes, multi-partner complex re-arrangements, read-through events in RNA-seq, and non-canonical re-arrangements (e.g. ITD and enhancer hijacking). Designing a sophisticated simulation data set which can generate performance statistics meaningful for real-time analysis of patient samples is a major endeavor beyond the scope of our current study.*

*As an alternative to simulation, we used paired tumor-normal DNA WGS data to determine the true positives of the predicted RNA-seq fusions, allowing us to assess the false positive rate of all the fusions predicted by CICERO and other methods. We feel that this method provides a more informative assessment that can guide real-life fusion analysis, which is superior to a simulation that does not account for the complexities in RNA-seq data generated from real patient samples. Our findings are shown in the newly added Figure 4d, shown above in our response to this reviewer’s first comment.*

Reviewer #2: This paper introduces a new method for gene fusion detection from RNA-seq data named CICERO. CICERO is a local assembly-based algorithm that integrates RNA-seq read support, identifies the candidate breakpoints, assembles the reads, maps the contigs and ranks the detected gene fusions after a fusion annotation process. This method is able to discover not only the canonical exon-to-exon chimeric transcripts, but also non-canonical fusions including internal tandem duplications, intronic and intergenic regions, etc.
To evaluate the performance of CICERO, the authors have used a dataset of 184 driver gene fusions discovered in 170 samples of various types of cancer. They have compared the performance of CICERO with four other existing tools namely ChimeraScan, deFuse, Fusion-Catcher, and STAR-Fusion. CICERO outperforms the other methods in terms of sensitivity and the ranking of the candidate fusions. They have also run CICERO on RNA-seq data from The Cancer Genome Atlas Gliobastoma Multiforme which includes 167 samples and resulted in detection of novel gene fusions.

The manuscript is well-written and the method is sound. I did not download the software or run it, so can't comment on that.

*[Response] We thank the reviewer for the great summary on CICERO’s algorithm and performance.*

I have two main comments:
1- Although "high false positives" is one of the motivations to develop this novel pipeline, only sensitivity and ranking of the driver fusions of the methods have been mentioned in the evaluation part of the paper. I believe the authors should provide tables/figures showing the specificity of the tools used for benchmarking.

*[Response] We thank the reviewer for this suggestion which was also raised by reviewer 1, who commented on the need to present the false positive (FP) rate of CICERO. A major challenge in assessing FP is the need to establish a truth data set. As discovery of somatically-acquired gene fusions is the main application for RNA-seq fusion detection, we decided to use RNA-seq fusions validated by somatic structural variations computed from paired tumor-normal DNA WGS as the truth data set for this analysis. This allows us to use DNA WGS as an orthogonal validation to determine the accuracy of fusions generated from RNA-seq. Among the 170 samples used for our benchmark analysis, 80 have matched tumor-normal whole-genome sequencing data, so we limited our FP analysis to this subset of the data. To ensure the sensitivity of somatic WGS SV calling, we took the union of somatic DNA SVs predicted by three algorithms (CREST, Manta and SvABA) for cross-validating predicted RNA-seq fusions. To account for the difference between a spliced RNA and its DNA coordinates, a predicted RNA-seq gene fusion is considered valid if both breakpoints are located within 100Kb of DNA somatic SV breakpoints computed by any of these three methods. By using this approach, we are able to generate the true positives for evaluating FP rate without relying on simulation or the “wisdom of crowd”.*

*In the revision, we incorporated this new analysis by 1) adding a new section “Assessing false positive rates in CICERO and other fusion prediction algorithms” in Methods; 2) revising the Results section “Comparison of CICERO with other methods of detecting diverse driver gene fusions” by changing the subtitle to “Comparison of CICERO with other methods of detecting somatic gene fusions” as the analysis has been expanded to all predicted gene fusions based on the review’s feedback. A new paragraph along with a new panel of Figure 4, Figure 4d, has been added to summarize the false positive rate of high-quality predictions as well all predicted fusions (shown below). This analysis also includes an additional method Arriba based on the feedback by Reviewer 2; 3) revising the title of the manuscript from “CICERO: an accurate method” to “CICERO: a versatile method” to better capture comprehensiveness of the analysis provided by CICERO; and 4) adding a discussion of high false positive rates to the third paragraph of the Discussion as follows:*

*“When comparing all predicted gene fusions to a truth dataset consisting of fusions cross-validated by somatic structural variations in matching WGS data (Methods), the overall false positive rate is high across all prediction programs, even for high-quality predictions from CICERO (78%), Arriba (82%), and STAR-fusion (91%). This suggests that knowledge-based filtering and manual curation are important steps in finalizing somatic gene fusions analyzed by tumor RNA-seq data.”*

*Figure 4. d) True positives (dark blue) and false positives (light blue) of predicted somatic fusions identified by CICERO and other fusion detection programs. The exact number of events is marked as (true positive/total prediction) under the name of each method. CICERO’s high-quality predictions are compared to those of STAR-fusion and Arriba (left panel) while all CICERO predictions are compared to those of FusionCatcher, deFuse, and ChimeraScan (right panel).*

2- As mentioned in this paper, a recent article titled "Accuracy assessment of fusion transcript detection via read-mapping and de novo fusion transcript assembly-based method" (<https://genomebiology.biomedcentral.com/track/pdf/10.1186/s13059-019-1842-9>) has assessed 23 methods for detection of gene fusions. From that study, STAR-Fusion, Arriba, and STAR-SEQR are reported as the most accurate and fastest for fusion detection on cancer transcriptome. The set of methods used here for benchmarking only include STAR-Fusion from the aforementioned assessment study. Given the findings in the benchmarking study, I believe the authors should also run Arriba and STAR-SEQR.

*[Response] We thank the reviewer for this suggestion and have attempted to run Arriba and STAR-SEQR on our benchmark data set. While the analysis using Arriba worked out well, we encountered major difficulties in completing the STAR-SEQR analysis after running the analysis for 30 days. Specifically, STAR-SEQR analyses for 100 out of the 170 benchmark samples were completed between 0.5-22hrs as expected while the remaining benchmark samples require either huge memory or very long run time. For example, one sample was killed after reaching 419 GB memory usage and another was completed after 19 days using 4 threads. We carried out a preliminary analysis using 119 benchmark samples which were successfully analyzed by STAR-SEQR after 30 days. As STAR-SEQR generates two fusion lists—a short list of fusions (named as STAR-SEQ) and a long list of fusions (named as STAR-SEQ-all), both results were incorporated in this preliminary analysis. As shown in the figure (Figure X, used only for response to this reviewer’s comment) below, the performance of STAR-SEQR and STAR-SEQR-all is far below the other methods in the categories of “highly expressed chimeric transcript” and “Non-canonical fusion transcript” while ITD events are not detectable. Given the incompleteness of STAR-SEQ as well as its general poor performance, we decided not to include the results from STAR-SEQR as part of our formal analysis of the benchmark samples.*

*Figure X. Comparison of performance of STAR-SEQR (shown in purple color) with the other methods on the analysis of benchmark samples. The results only included 119 benchmark samples which were successfully analyzed by STAR-SEQR*

*Results from Arriba were incorporated into our revised Figure 4, showing its sensitivity and false positive rate along with those of other methods. We noticed that Arriba generates high, medium and low confidence predictions. Similar to a previous report by Haas et al, we noted that incorporating all predicted fusion by Arriba will generate the best performance statistics for this tool as shown in Figure S10 below, and so we presented all predicted fusions by Arriba in our analysis. Specifically, the performance of Arriba is comparable to CICERO in the categories of “highly expressed chimeric transcript” and “lowly expressed chimeric transcript”. However, Arriba has much lower sensitivity in predicting “Non-canonical fusion” and cannot detect any ITD events. On the subject of false positives, Arriba has a slightly higher false positive rate (82%) compared to the high-quality predictions from CICERO (78%).*

*Figure S10 Comparison of the sensitivity (top panel) and ranking (bottom panel) of the driver fusions detected by HQ versus all Arriba prediction. Fusion genes are shown in the four categories of highly expressed chimeric transcript, low-expressed chimeric transcript, internal tandem duplication (ITD), and other non-canonical fusions involving intronic or intergenic regions.*

**Second round of review**

**Reviewer 1**

The authors did a great job in addressing my concerns. I have no further comments.

**Reviewer 2**

The authors have responded to all my comments. I completely understand the issue of not being able to run STAR-SEQR. About changing the method name, I sympathize with the authors, but the problem is that capitalization won't help distinguish methods in a web search, etc.
